# Ultra Processed Food Consumption in Children and Adolescents: Main Food Group Contributors and Associations With Weight Status

**DOI:** 10.1111/nbu.70001

**Published:** 2025-04-01

**Authors:** Evgenia Petridi, Emmanuella Magriplis, Kalliopi Karatzi, Evelina Charidemou, Elena Philippou, Antonis Zampelas

**Affiliations:** ^1^ Department of Life Sciences, School of Life and Health Sciences University of Nicosia Nicosia Cyprus; ^2^ Department of Food Science and Human Nutrition Agricultural University of Athens Athens Greece; ^3^ Department of Biological Sciences University of Cyprus Nicosia Cyprus; ^4^ Department of Nutritional Sciences King's College London London UK

**Keywords:** adolescents, children, NOVA classification system, obesity, overweight, ultra‐processed foods (UPF)

## Abstract

Consumption of ultra‐processed foods (UPFs) is thought to play an important role in the development of childhood obesity worldwide. The aim of this study was to assess the main food groups contributing to UPF consumption and their association with weight status. Following exclusion of children with implausible intakes and misreporters, the final sample included 443 of 484 eligible (children and adolescents aged 2–18 years old) (91.5%), from the *Hellenic National Nutrition and Health Survey* (*HNNHS*). UPF items reported in 24‐h recalls were identified according to the NOVA4 system and the proportion of their contribution to the daily energy intake was calculated. Main UPF food contributors were derived for the total population and by weight status. The association between weight status and UPF intake for the main contributors was examined using generalised linear models. The average percentage of total daily energy provided by UPFs was 39.8%. Four major food groups were found to contribute > 10% of total UPF intake: ready‐to‐eat/heat dishes (36.2%), sweet grain products (21.4%), savoury snacks (15.4%) and sweets (12.9%). These provided 86% of the total UPF intake, with no significant differences between children's weight status. There was also no significant association between the total percentage of energy as UPF and obesity. The relatively high contribution of UPFs, however, to children's daily energy intake in Greece emphasises the need for public food awareness campaigns for health promotion. Evaluation of the contribution of different food groups and not only of total UPF intake is also important.

## 1 Introduction

Increasing evidence suggests that the population of southern Europe is transitioning away from traditional eating habits, such as the Mediterranean diet, in favour of more ‘Westernised’ eating patterns Grosso et al. (2014). These modern dietary habits tend to promote unhealthy behaviours related to weight gain, including high consumption of readily available, palatable foods with low nutritional value Pereira‐da‐Silva et al. (2016) and an increased reliance on ultra‐processed food (UPF) Vedovato et al. (2021). The latter foods are designed to be consumed on the go and frequently without utensils. They are sold as ready‐to‐eat dishes, snacks and drinks and usually displace homemade meals. Furthermore, the techniques and the additives used to produce them have been suggested to make them highly palatable, increasing the tendency of overeating and cravings and affecting appetite and satiety regulation Schulte et al. (2015). Consequently, this may lead to overconsumption, especially of foods rich in simple carbohydrates and fat, resulting in weight gain Hall et al. (2019) and obesity.

Obesity has become a global epidemic: in 2022, 43% of adults aged 18 years and over were overweight and 16% were living with obesity World Health Organization (2024). The number of children affected by obesity has also increased by tenfold in the past four decades World Health Organization (2017). This is of great importance since childhood obesity is associated with an increased risk of obesity later in life Ward et al. (2017) and early onset of adverse cardiometabolic health conditions Chung et al. (2018). Increasing evidence suggests that the key risk factor for overweight and obesity is unhealthy dietary habits and eating contexts Sirkka et al. (2021; Neves et al. 2022) and behaviours found to be associated with a high percentage of UPF intake Neves et al. (2022). Furthermore, ad libitum UPF intake was reported to result in higher energy intakes compared to intake of their unprocessed counterparts Hall et al. (2019).

Recent studies have shown that high consumption of UPFs is associated with a higher prevalence of obesity in adults (Juul et al. 2018; Rauber et al. 2021) and in children (Menezes et al. 2023; Costa et al. 2018), although evidence remains controversial (Enes et al. 2019; Oliveira et al. 2020). Despite the controversy, the concern is that the majority of UPFs contain little or no whole food, and a significant number are of very low nutritional value and contain a high content of energy, sugars, saturated fats (Menezes et al. 2023), factors linked to chronic disease (Rauber et al. 2018). UPF intake has been associated with increased risk of arterial hypertension (Mendonça et al. 2017), metabolic syndrome (MetS) in adolescents (Tavares et al. 2012) and abnormalities in the lipid profiles in children (Rauber et al. 2015). The most widely used method to assess the degree of food processing is the NOVA classification system (Monteiro et al. 2019). Many studies using this method found that UPF contributes about 50% to the total energy intake in children and adolescents (Araya et al. 2021; Moubarac et al. 2017; Rauber et al. 2018). However, it remains controversial whether the degree of processing alone makes a difference or whether the nutrient content, the meal patterns or the overall food matrix is of more importance.

Specifically, a prospective study in children showed that those consuming a specific dietary pattern diet, characterised as high in white bread, crisps and sugary drinks, was significantly associated with increased risk for childhood overweight at 10 years, compared to a minimally processed dietary pattern high in vegetables, sauces, rice/pasta and savoury dishes (Sirkka et al. 2021). The authors used Principal Component Analysis to determine their population's dietary pattern and not the NOVA classification, and described the minimally processed pattern, the one characterised by whole grains and vegetables, despite it also including the presence of sauces as well. Most studies provide data mostly related to total UPF intake and contribution to daily energy intake, through NOVA classification of foods in the diet, although the proportion of the main food contributors to UPF intake may be of great importance as well as shown by a recent systematic review and meta‐analysis in adults (Mendoza et al. 2024). This type of analysis may shed light on these controversial findings.

Therefore, the present study is to our knowledge, the first study in Greece that aims to assess the proportion of UPFs in daily energy intake and identify the main food groups contributing to UPF consumption among children and adolescents, utilising data from the *Hellenic Nutrition and Health Survey* (*HNNHS*). The association between total UPF intake, specific UPF food types and weight status, was also explored.

## 2 Methods

### 2.1 Study Design and Population

The *HNNHS* is a population‐based study designed to assess the health and nutritional status of Greek children and adults. It was carried out between September 2013 and May 2015. A nationally representative sample was selected with a random stratified design based on the 2011 census data. The study's details have been published elsewhere (Magriplis et al. 2019). Trained interviewers using the Computer Assisted Personal Interview (CAPI) method, collected data on anthropometric, sociodemographic and lifestyle parameters with an in‐person interview at the participant's residence. A written consent form was provided by the legal guardian of all children and adolescents.

For the purpose of this study, all children and adolescents aged 2–18 years (Children: 2–11 years and Adolescents: 12–18 years) (from now on referred to as children) who had provided at least one 24‐h dietary recall were primarily included (*n* = 733). Exclusion criteria included implausible intakes set at ≥ 6000 and ≤ 600 kcal (*n* = 15), and mis‐reporters assessed using Goldberg's equations, as modified by Black (Black 2000). This methodology is based on the ratio of reported Energy Intake and the estimated Basal Metabolic Rate (BMR) as per Schofield's age‐ and sex‐specific equations (Koletzko et al. 2005), multiplied by a specific standard of Physical Activity Level. A total of 172 children were classified as over‐reporters and 62 were classified as under‐reporters and therefore removed from the dataset leading to a sample size of 484 children. Details regarding calculations of over‐reporters and under‐reporters have been published (Mitsopoulou et al. 2020). Furthermore, although missing anthropometric data were imputed, in cases where children had missing information for both weight and height, no imputations were performed in order to minimise bias, and these children were excluded (*n* = 41). More specifically, missing data on weight and height were imputed by modelling the variable distribution by age (for each child with missing information on weight or height), under the assumption of Missing at Random (MAR) upon first screening. Imputed data were checked to ensure they are reasonable compared to the observed non‐missing data distribution. MAR was followed since data were preliminarily checked during the cleaning process and no pattern between missing weights and specific disabilities regarding the status of the main responder was found (see Data S1 online). Further information is provided in the Data S1 uploaded. The final sample included in this study was 443 children (91.5% of children with plausible intakes).

### 2.2 Data Collection

#### 2.2.1 Dietary Assessment

Assessment of dietary intake was done through a 24‐h recall (24hR) by trained interviewers using the Automated Multi‐pass Method (AMPM) (Blanton et al. 2006). Two 24hR were conducted on non‐consecutive days, the first in‐person and the second over the telephone 8–20 days later. Dietary data were collected from parents who were used as proxies for children < 12 years. Adolescents ≥ 12 years old completed the dietary recall by themselves. Portion sizes were estimated by using age‐specific food atlases and household measures (e.g. cups, glasses, spoon sizes). Energy and nutrients for each food were derived from the Nutrition Data System for Research (NDSR) (developed by the University of Minnesota) (University of Minnesota 2024) and the Greek food composition tables for traditional Greek recipes (e.g. baklavas) (Trichopoulou and Georga 2004).

The total daily energy intake (TEI) of NOVA 4 food classified foods was calculated by summing the energy content of each food item and then the average intake per child per day was estimated.

#### 2.2.2 Food Grouping and Nutrient Intake Estimation

Food and beverage items were classified according to the NOVA 4 food classification system. NOVA is a system that categorises food into 4 groups based on the degree and purpose of processing each food has undergone. Specifically, NOVA1 includes unprocessed or minimally processed foods, NOVA2 includes processed culinary ingredients, NOVA 3 contains processed foods and NOVA 4 foods that have encountered a high degree of processing not regularly performed at home such as refining, hydrolysation and pre‐frying (Monteiro et al. 2019; Khandpur et al. 2021). For this study foods in NOVA 4, usually defined as ultra‐processed, were evaluated.

A four‐step process was performed to identify UPFs. In the first step, a list of all food consumed by each child was compiled from the 24hRs. In Step 2, UPF foods were identified and categorised as NOVA 4 following the procedure of gathering information (brands where possible) from 24hRs, the eating location (bakeries compared to home and other food‐specific facets). At this step, foods that were of unclear classification were flagged and were categorised following team consultation. For the decision to be made, reported food details from the child's 24hRs were evaluated (where it was consumed, if a recipe was homemade or ready to eat, etc.). In Step 3, all foods were categorised into specific food groups based on their main food ingredient and then grouped into eight larger categories. For example, the food group, ‘breakfast cereals’ contains all reported cereals that are refined or whole grain but contain added sugar, hence their being categorised in a separate food group other than the main food group ‘grains and cereals’. Details of the food groups created can be seen in Table 1. In Step 4, the energy of all food per food group was summed, for each recall (in kcal, as per each food nutrient profile) and was then averaged for children with two 24hRs. The energy derived from all food groups of NOVA 4 was summed and the value was divided by the total mean daily energy intake of each child to calculate the total proportion of the diet consisting of UPFs. Food group contribution was derived as percent total energy per food group (kcal) divided by the total energy (kcal) from all NOVA 4 food groups consumed by each child and then multiplied by 100 (Block et al. 1985). Proportions were calculated for the total sample and by children's weight status groups.

**TABLE 1:** NOVA 4 Food Group derivation with types of food and beverages included in each group.

NOVA 4 Subgroups	Foods and beverages included in each subgroup
Ready to eat/heat dishes	
Ready‐to‐eat/heat sandwiches	Hotdogs, wheat Arabic pita, crepes, baguettes, sandwiches, tortillas, toast
Ready‐to‐eat/heat pizza	Pizza
Savoury pies/tarts	Pies (cheese, ham, sausage, vegetables)
Other ready‐to‐eat/heat‐mixed dishes	Stuffed pasta, Greek meat or poultry (souvlaki), meat products (schnitzel, gyros)
Flavoured dairy products	
Milk	Condensed sweetened milk
Yogurt	Flavoured yogurt
Flavoured dairy drinks	Milkshake, hot chocolate
Sugar‐sweetened beverages	
Sugar‐sweetened and diet soft drinks	Sugar‐sweetened and diet soda, iced tea
Fruit drinks	Fruit juices with added sugars or concentrated fruit remixed with water
Branded breads	
Grain products	Sliced bread
Pita breads	Arabic pita, pita bread with additives, tortillas, sesame bread rings
Savoury snacks and baked goods	
Savoury snacks	Salted crackers, breadsticks, rice cakes, sesame bread rings with cheese, Cheetos, chips (potato, tortillas), popcorn, bread with cheese
Baked goods	Butter croissant, short & puff pastry savoury pies (e.g. cheese pie, spinach pie, meat pie, etc.)
Sweet grain products	
Bakery sweet products	Chocolate croissants, short & puff pastry sweet pies, ‘tsoureki’, baklava, ‘melomakarono’
Cereal bars and biscuits	Bars, biscuits
Waffles and crepes	Waffles, crepes
Breakfast cereals	Cereals (wheat, whole grain, oat) with added sugar
Sweets	
Desserts	Gelatin desserts, traditional sweets with sirup, sweets with chocolate, puddings (chocolate, vanilla), mille‐feuille, loukoumi, cakes, cheesecake, profiterole, ice‐creams, halva (sesame), Honey sesame bars, chocolate‐pie
Sweet pies and tarts	Pies (lemon pie), sweet tarts
Candies & chocolate bars	Candies, jellies, chewing gums, chocolate, chocolate waffle bars, syrups, jams
Other sweet UPFs	Sweet spreads (praline, sesame, peanut butter)
Other	
Fast‐food or reconstituted meat, poultry and fish products	Ham from reconstituted meat or poultry, bacon, meat (pate), sausages, nuggets (chicken, cheese)
Fast food or pre‐prepared potato products	Fast food, pre‐prepared, frozen French fries
Fats, spreads and sauces	Margarine (with or without butter), cheese spread, sauces, dips, cream cheese
Other UPFs	Distilled alcoholic drinks, sparking water, chocolate powder, baby formulas (milk, creams, chamomile formula)

Abbreviation: UPFs, ultra‐processed foods.

### 2.3 Anthropometric and Other Variables

#### 2.3.1 Weight Status Assessment

Body Mass Index (BMI) was used to evaluate the children's weight status. Bodyweight and height were measured by the parent or guardian for children < 12 years and for adolescents ≥ 12 years by themselves. Height and weight measurements were also performed by researchers in a randomly selected subsample of the population (*n* = 88), five of which were aged < 2 years and therefore not included in the current study, resulting in a subsample total of *n* = 83 children A total of 107 data were imputed for children when either height or weight data was available, using the Missing at Random (MAR) process (information stated previously). BMI was calculated by dividing weight in kg by height in metres squared (kg/m^2^). To categorise children and adolescents according to their BMI, the extended International Obesity Task Force (IOTF) tables were used (Cole and Lobstein 2012). These tables are a set of growth reference charts and cutoffs, that consider age and sex differences in growth patterns, specifically designed to monitor and address childhood obesity on a global scale. Children who were overweight and children living with obesity were grouped for further analysis to have adequate power of analysis.

#### 2.3.2 Other Variables

Demographic, lifestyle and socio‐economic data such as sex, age, physical activity, screen time for children parental educational level and employment status were collected. Physical activity was evaluated using validated age‐specific questionnaires (i) Preschool‐aged Children Physical Activity Questionnaire (Pre‐PAQ Home Version) (Dwyer et al. 2011) for children 2–12 years old (Timmons et al. 2007) and (ii) the International Physical Questionnaire—Adolescents (IPAQ‐A) for those 12–18 years old (Hagströmer et al. 2008). Similarly, the IPAQ‐A is a 7‐day recall tool that estimates total metabolic equivalents (METs) based on data collected on activities carried out at school (formal physical education lessons and recess/break times), at home, at leisure time and during transport/journeys. The physical activity level was estimated by multiplying the time spent on each activity by the corresponding MET‐value of the specific activity, using published values (Arvidsson et al. 2005). Screen time was defined as the average time spent in front of any type of screen per week, not including active gaming and a mean daily average was estimated. Parental education level was categorised by school years (elementary school, middle school and higher level of education) and their employment status was categorised as employed, unemployed (including homemakers) or retired.

### 2.4 Statistical Analyses

The proportion of total daily energy intake from UPFs was estimated according to NOVA 4 food groups and their specific food subgroups. P–P plots were used to assess the normality of the distribution. Numerical variables following normal distribution were presented with a mean (standard deviation) and those that were skewed were presented as medians and range (P25 and P75). Categorical variables were described as relative frequencies. Between‐group differences were estimated with parametric (student *t*‐test) or non‐parametric methods (Mann Whitney *U*‐test) for numerical and with chi‐square test for categorical data. A general linear model (GLM) for maximum logistic likelihood analysis was used, of the binomial family, to assess the likelihood of being overweight/living with obesity compared to normal weight according to dietary contribution of % total energy intake (%TEI) of NOVA 4 food group, categorised in tertiles. The model was adjusted for age (continuous by 4‐year increase), sex (binary), the area of residence (main metropolitan areas, islands & Crete, mainland), total screen time (continuous) and %TEI. The predicted probability of overweight and obesity was also examined only for the main NOVA 4 food group contributors using a continuous approach, to examine the level of intake at which they may have an effect on children's weight status. A subsample of all children was weighed and measured by the researchers and a high correlation (> 0.95) was found. More specifically a random sample of 83 children were weighed and the mean weight measured was 41.8 ± 19.3 while the mean weight reported by the same children/guardians was 40.6 ± 18.6. The correlation for height was comparative to that of weight. Outcomes are presented according to corresponding 95% confidence intervals. Significance was set at alpha 5% (*p* < 0.05) being considered statistically significant. The statistical software package STATA 17.0 was used for analysis (Stata Corp LLC., College Station, TX, USA).

## 3 Results

The children's anthropometric, lifestyle and sociodemographic characteristics are shown in Table 2. Among 443 for which weight and height data were available or reported (336 and 107 respectively), 75% (*n* = 332) were categorised as normal weight and 25% (*n* = 111) as overweight/living with obesity. Between groups, significant differences were found only for screen time with overweight children/children living with obesity (27%) having a 2.5‐h median screen time (27%) compared to normal‐weight children (73%) who had a 2.2‐h median screen time, although in both cases it was above the daily 2‐h level recommended by the American Paediatric Association (Daniels et al. 2015). Most primary guardians were employed, and their average level of education was more than 12 years, with no distribution differences by weight status observed.

**TABLE 2:** Anthropometric and lifestyle characteristics of children and adolescents and guardians' sociodemographic information.

	Total	Normal weight	Overweight/Obesity	*p*
Total sample, *n* (%)	443	332 (74.9)	111 (25.1)	
Αge (years), (median, [range])	9 (6, 14)	9 (5, 14)	9 (6, 12)	0.176
Weight (kg), (median [range])	36 (21, 55)	32.7 (19, 52)	42 (28.5, 65)	0.000
Height (m), mean (SD)	1.4 (0.3)	1.4 (0.3)	1.4 (0.2)	0.451
ΒΜΙ (kg/m^2^), mean (SD)	18.7 (4.0)	17.4 (3.0)	22.6 (4.2)	0.000
Group Age, *n* (%)				
Children	258 (58.2)	190 (57.2)	68 (17.8)	0.456
Adolescents	185 (41.8)	142 (42.8)	32 (7.2)	
Sex, *n* (%)				
Male	221 (49.9)	167 (37.7)	54 (12.2)	0.763
Female	222 (50.1)	165 (37.2)	57 (12.9)	
Total screen time (hours), (median, [range])	2.2 (1.2, 3.5)	2.2 (1.12, 3.5)	2.5 (1.5, 3.5)	0.024
Primary Guardian Education level, *n* (%)				
≤ 6 years	12 (3.8)	10 (3.2)	2 (0.6)	0.303
6–12 years	124 (39.6)	86 (27.5)	38 (12.1)	
≥ 12 years	177 (56.5)	135 (43.1)	42 (13.4)	
Primary Guardian Professional Status, *n* (%)				
Employed	216 (70.4)	159 (51.8)	57 (18.6)	0.849
Unemployed/Homeworkers	76 (24.8)	57 (18.6)	19 (6.2)	
Pension	15 (4.9)	12 (3.9)	3 (1.0)	

*Note:* *p* < 0.05; Student *t*‐test for normally distributed values and Mann–Whitney test for skewed numerical variables (two group comparison); chi‐square test for categorical variables.

Abbreviation: IQR, Interquartile range.

Table 3 shows the percentages of total energy intake from NOVA 4 food group that were consumed in total and by normal weight and overweight children/children living with obesity. Νο significant differences were observed between the groups; for the total population the proportion was 39.8%, for normal weight and overweight children/children living with obesity the percentages of total energy intake from NOVA 4 food group were 39.5% and 40.2%, respectively. Moreover, differences in percentage of total energy intake contribution from NOVA 4 food subgroups between normal weight and overweight children/children living with obesity were not statistically significant, except from the subgroup of ‘other’ 2.1% (which includes the food subgroups reconstituted meats, pre‐prepared potatoes, spreads and sauces distilled alcoholic drinks, sparkling water, chocolate powder). Consequently, normal weight children eat more reconstituted meats, pre‐prepared potatoes, fats, spreads and sauces than overweight children/those living with obesity.

**TABLE 3:** Percent of NOVA 4 foods consumed (as total and by subgroup) with mean energy intake; results presented for all children and by children's weight status.

%E NOVA foods, median, (range)	Total	Normal weight	Overweight/Obesity	*p*
NOVA 4	39.8 (25.4, 55.0)	39.5 (24.5, 54.3)	40.2 (27.0, 57.3)	0.535
Ready to eat/heat dishes^a^	22.9 (14.2, 35.5)	22.1 (13.7, 35.5)	26.2 (16.7, 36.9)	0.197
Flavoured dairy products^b^	4.3 (2.0, 8.3)	3.02 (1.6, 7.2)	6.6 (3.5, 10.7)	0.183
Sugar‐sweetened beverages^c^	3.0 (1.8, 5.0)	3.5 (1.9, 5.1)	2.6 (1.3, 3.4)	0.20
Branded breads^d^	6.3 (3.6, 10.1)	6.62 (4.0, 10.2)	4.6 (3.3, 9.9)	0.409
Savoury snacks^e^	13.0 (5.7, 24.0)	12.6 (5.7, 23.2)	13.4 (8.4, 26.8)	0.504
Sweet grain products^f^	10.8 (5.8, 19.5)	10.8 (5.8, 19.4)	10.8 (5.6, 19.7)	0.98
Sweets^g^	8.5 (4.2, 13.9)	8.3 (4.1, 13.7)	9.93 (4.5, 14.2)	0.959
Other^h^	2.1 (0.7, 7.0)	2.5 (0.8, 7.4)	0.9 (0.5, 4.0)	0.02

*Note:* *p* < 0.05; Mann–Whitney test for skewed numerical variables (two group comparison); %E: total daily energy intake; Range: 25th–75th percentiles of the distribution.

^a^ Sandwiches, pizza, pies, tarts, mixed dishes.

^b^ Milk, yoghurt, drinks.

^c^ Sugar‐sweetened drinks, fruit drinks.

^d^ Grain products, pita bread.

^e^ Savoury snacks, bakery pies.

^f^ Bakery sweets, cereal bars and biscuits, waffles and crepes, cereals.

^g^ Desserts, sweet pies and tarts, candies, jams, other sweet UPFs.

^h^ Reconstituted meats, pre‐prepared potatoes, fats, spreads and sauces, other ultra processed foods (distilled alcoholic drinks, sparkling water, chocolate powder, baby formulas).

**FIGURE 1:**
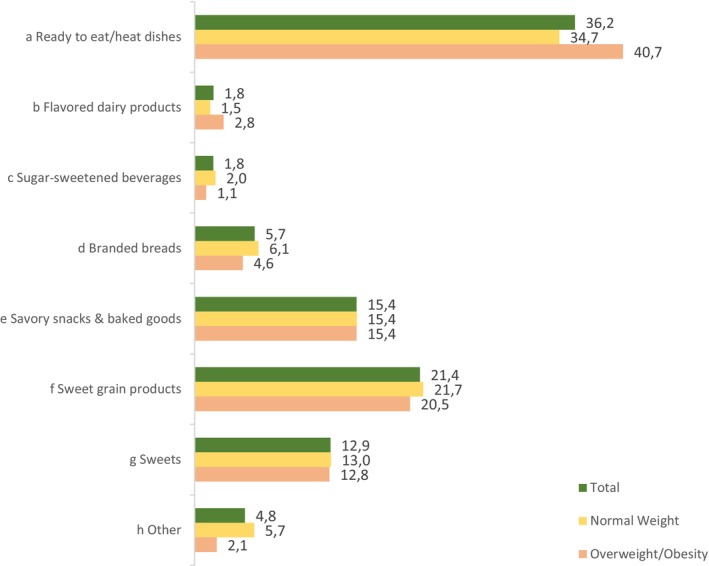
Main food groups contributors to NOVA 4 category in children and adolescents; results depicted by weight status. Values derived as percent total energy per food group (kcal) divided by the total energy (kcal) from all NOVA 4 food groups consumed by each individual, and then multiplied by 100. ^a^Sandwiches, pizza, pies, tarts, mixed dishes, ^b^Milk, yoghurt, drinks, ^c^Sugar‐sweetened drinks, fruit drinks, ^d^Grain products, pita bread, ^e^Savoury snacks and baked goods, ^f^Bakery sweets, cereal bars and biscuits, waffles and crepes, cereals, ^g^Desserts, sweet pies and tarts, candies, jams, other sweet UPFs, ^h^Reconstituted meats, pre‐prepared potatoes, fats, spreads and sauces, other UPFs (distilled alcoholic drinks, sparkling water, chocolate powder, baby formulas).

To increase understanding of specific food group intake, the percentage of each (sub)‐food group of UPF to total energy provided by all NOVA 4 food groups was calculated. These are presented in total and by weight status in a bar graph (Figure 1). Four food groups contributed > 10% to total NOVA 4 adding up to 86% of total UPF intake. Ready‐to‐eat/heat dishes are the most prominent with 36.2% of the total population (34.7% among normal weight children and 40.7% among overweight children/children living with obesity) followed by sweet grain products (21.4%), savoury snacks and baked goods such as savoury pies with cheese, spinach or meat sweets in contrast to sweet grain products such as short & puff pastry sweet pies or baklava (15.4%) and sweets (12.9%).

The adjusted Odd Ratio (AOR) between weight status and tertile of NOVA 4 consumption (as % of total caloric intake) is depicted in Table 4. No significant differences were found by tertile of NOVA 4 intake as a proportion of calories. The model was adjusted for sex, age category, total energy intake and total screen time. The same analysis was conducted using non‐imputed weights, and no differences were found.

**TABLE 4:** Multiple logistic regression between weight status and ultra‐processed foods consumption (%kcal/day) in children of the Hellenic National Nutrition and Health Survey (HNNHS).

Weight status^a^	Odds ratio	(95% confidence interval)*	
NOVA 4^b^, (%kcal/day) (median, range of first tertile: 19.5 [13.2–25.5])	—	—	—
2nd Tertile (38.6 [33.9, 44.4])	1.6	0.92	2.84
3rd Tertile (60.8 [54.6, 72.7])	1.0	0.56	1.91

*Note:* Results following logistic regression by tertile of NOVA 4 (based on % energy contribution) and adjusted for sex, energy intake, age category (Children: 2–11 years and Adolescents: 12–18 years) and total screen time. NOVA 4 tertile distributions between children and adolescents did not differ (*p* for all tertiles > 0.1).

^a^ Weight status was normal weight and overweight participants/participants living with obesity.

^b^ Reference tertile for % energy NOVA 4 was the 1st tertile.

Significant at a = 5%.

**FIGURE 2:**
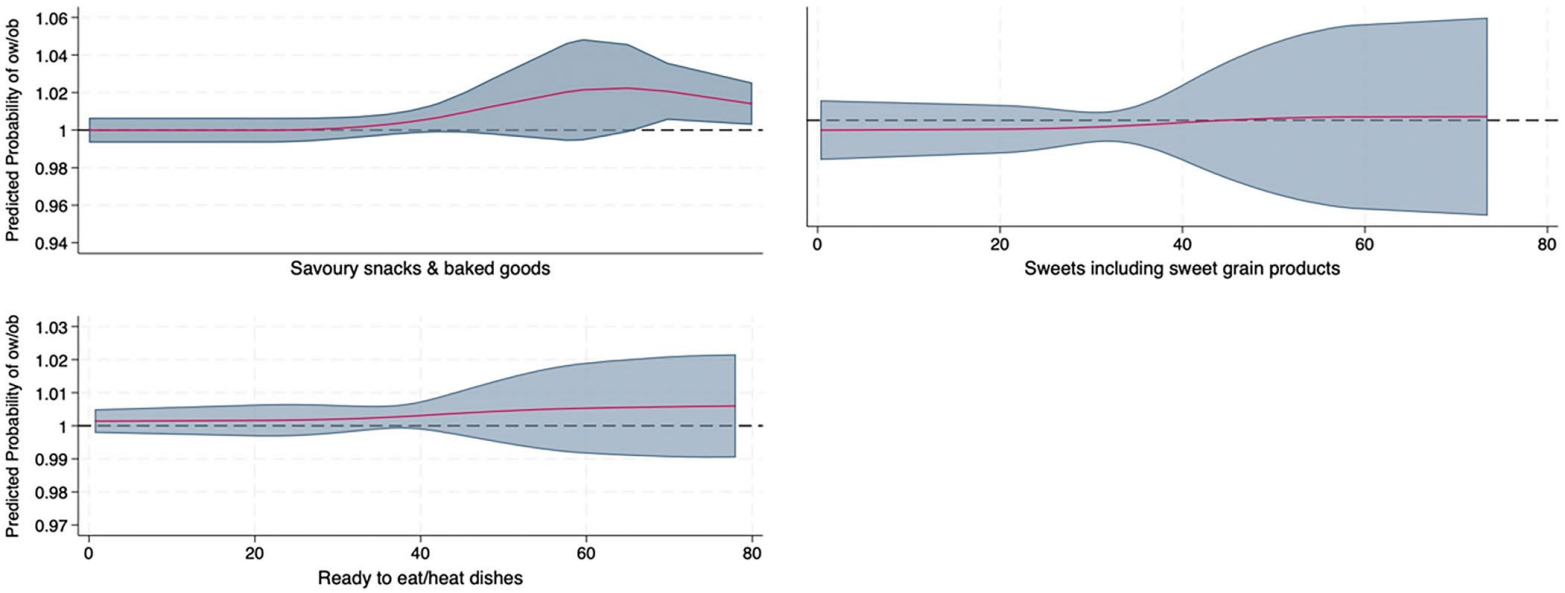
Predictive probability (margins) of overweight or obesity among children by specific NOVA 4 food group intake (% of total energy). Cubic spline graphs based on generalized linear models (GLM) of the binomial family and logit link; adjusted for sex, age, mean daily energy intake and area of residence. The three main NOVA 4 contributors are depicted (NOVA4 group seen on the *x*‐axis for each graph displayed). The vertical dotted line represents no effect. The red line represents the measure of effect (Predicted odds of overweight/obesity by an increased level of energy‐adjusted consumption). Savoury snacks and baked goods: salted crackers, breadsticks, all crisps and Cheetos, short crust and puff pastry type savoury pies Ready‐to‐eat/heat dishes: Sandwiches, pizza, pies, tarts, mixed dishes, Sweets include sweet grain products: Desserts, sweet pies and tarts, candies, jams, bakery sweets, cereal bars and biscuits, waffles and crepes and breakfast cereals.

Lastly, specific food groups highly contributing to NOVA 4 as a percentage of total energy intake were modelled in relation to the probability of overweight and obesity (Figure 2). The results showed that the only food group that was associated with increased probability of being overweight/obese was savoury snacks and baked goods for children and adolescents who consumed these as > 62% of their total daily energy intake, potentially indicating an effect on weight status over and above this level of consumption. However, the percentage of children found to exceed this level was small (0.4%, *n* = 2). The total proportion of all NOVA 4 subgroups was not significantly associated with weight in accordance with logistic regression results (see Table 4).

## 4 Discussion

The current study aimed to assess the proportion of UPFs in the daily energy intake of a sample of Greek children, using the NOVA food classification system. Additionally, the study sought to identify the primary food groups contributing to UPF intake and examine their association with the likelihood of overweight and obesity. The primary finding of this investigation was the prevalence of a relatively high UPF diet among most Greek children, with UPFs accounting for 39.8% of their total daily energy intake. However, no significant association was found between the total percentage of energy as UPF and obesity, suggesting that the major contributors to UPF intake should be further evaluated. Four food groups collectively contributed over 80% of total UPF intake, specifically sweets, sweet grain products, savoury snacks and baked goods and ready‐to‐eat/heated dishes. Notably, only the consumption of savoury snacks and baked goods was statistically associated with an increased likelihood of overweight and obesity when it exceeded 62% of total caloric intake. However, this last observation needs further investigation, due to the very small number of children (*n* = 2) consuming this level of savoury snacks and baked goods. A similar trend was observed for ready‐to‐eat/heat dishes, though statistical significance was not reached.

The findings from this study align with existing research indicating a relatively high prevalence of UPF consumption among children (Juul et al. 2022; Rauber et al. 2018; Moubarac et al. 2017; Araya et al. 2021; Detopoulou et al. 2023). In particular, the reported range from studies conducted in North America was from 47.7% of total energy (Moubarac et al. 2017) to 57% (Juul et al. 2022) with the latter study also reporting an increase in average UPF intake in the population over a 16‐year period (from 53.5% of kcal in 2001–2002 to 57% of kcal in 2017–2018) (Juul et al. 2022). A cross‐sectional study from the UK *National Diet and Nutrition Survey (NDNS)* (2008–2014) population aged 1.5 years or over, revealed that UPF accounted for 56.8% of their daily energy intake (Rauber et al. 2018). Subsequent research indicated the highest levels of UPF consumption among children aged 4 to 10 years were also recorded in the UK, according to data from the *NDNS* (Onita et al. 2021), with these foods comprising an estimated 65% of their daily caloric intake. Another study conducted in Greece found that 40.7% of the daily caloric intake was derived from UPFs (Detopoulou et al. 2023), among a convenience sample of young adults (specifically university students aged 18–22 years old). However, no other studies in Greece have evaluated UPF intake in children.

This study did not find a significant association between UPF consumption and obesity in children, which is consistent with some previous studies (Enes et al. 2019; Oliveira et al. 2020). Interestingly in the study of Enes et al., although UPF consumption contributed 50.6% of the total calories consumed, which is higher than what was found in our study, no associations with anthropometric indices were observed. In the study of Oliveira and his colleagues, the contribution of UPF consumption to total energy consumption was 43.7%. This value is much closer to the value found in the present study. In contrast, other studies have found that higher consumption of UPFs is associated with an increase in the risk of overweight/obesity (Neves et al. 2022; Vedovato et al. 2021; Neri et al. 2022). More specifically, a Brazilian cross‐sectional study among 790 adolescents aged 14–19 years old observed that UPF consumption was higher among adolescents who had an inappropriate eating context regarding the habits of eating out for these meals, eating in noisy places or in bed, standing or walking, in front of screens and without company and was associated with an increased likelihood of obesity (Neves et al. 2022). The authors did not assess the actual amount but only the frequency of consumption. Furthermore, in Portugal, a longitudinal study with 1175 children showed that higher consumption of UPFs at 4 years of age was associated with a higher BMI *z*‐score at 10 years of age (Vedovato et al. 2021), in agreement with another prospective study conducted in 3 year‐olds in The Netherlands (Sirkka et al. 2021), even though the latter examined a UPF type diet based on principal component analysis and not on NOVA system categorisation. A combination of the NOVA system with dietary pattern derivation from a food frequency questionnaire was used by Neri et al., in adolescents and found that higher consumption of UPFs was associated with 52% and 63% higher odds of abdominal and visceral overweight/obesity, respectively, compared to lower consumption (Neri et al. 2022). Results were based on the proportion of the population following a UPF‐like dietary pattern and not on the proportion of UPF in the total daily energy intake.

A large proportion of UPF are nutritionally unbalanced Moubarac et al. (2013) and characterised by a high density of energy, sugars, saturated and *trans* fats and sodium and a low content of protein, fibres and micronutrients Rauber et al. (2018); Araya et al. (2021); Costa de Miranda et al. (2021). Hence, the main problem that may explain associations between high UPF intakes and poor health outcomes reported in other studies is their potentially low nutritional value which fails to meet the WHO recommendations for the prevention and control of obesity Moubarac et al. (2013). However, some UPF have been found to have ‘healthier’ front of pack labelling scores with regards to nutritional composition, although they remain higher in energy, total sugar, salt and saturated fats compared to their minimally processed counterparts Dicken et al. (2024). It is noteworthy that children and adolescents with normal‐weight preferred sweet grain products, sweets and sugar‐sweetened beverages, as well as bakery products compared to overweight children/children living with obesity who reported a preference towards foods such as ready to eat/heat dishes, flavoured dairy products and savoury snacks. Although we found no association between UPF consumption and childhood obesity, based on the nutritional content and potential quality of UPF foods, and associations with health status later in life, the high consumption of foods high in added sugar, salt and saturated fats is a potential public health concern (Cordova et al. 2023; Moubarac et al. 2013; Oliveira et al. 2020; Rauber et al. 2015).

One possible mechanism to explain an association between UPF consumption and obesity which has been proposed is that their high‐intensity flavouring makes them extremely palatable and less satiating than less processed foods and may even override endogenous satiety mechanisms (Neves et al. 2022). This makes them very appetising but less filling compared to meals that are less processed. This can result in excessive consumption of food (Hall et al. 2019) and interfere with the body's normal signals of fullness, making it more difficult for individuals to cease eating once they are satisfied. Moreover, UPFs generally have a higher caloric density and lower fibre content, which can lead to overeating and unhealthy eating patterns (Juul et al. 2018). Finally, it has been suggested that large portion sizes, ubiquitous availability and affordability of UPFs encourage constant snacking and unintentional overeating and may displace less processed and healthier foods from the diet (Moubarac et al. 2017). In addition, other studies have also found that higher UPF consumption per daily energy intake is associated with not only the risk of obesity (Juul et al. 2018; Rauber et al. 2021) but also of chronic diseases, such as type 2 diabetes (Levy et al. 2021), dyslipidemias (Rauber et al. 2015) and hypertension (Mendonça et al. 2017).

Overall, the type of UPF consumed and the total dietary pattern need to be examined, rather than the total consumption of UPF alone. For example, in Greece higher intakes of free sugars were associated with two different meal patterns; one characterised by higher consumption of sweets, sugar‐sweetened beverages (SSBs), fast food and fries and another consisting of whole fruits, 100% fruit juice, vegetables, legumes and honey/jam spreads (Magriplis et al. 2021). The study examined the association of added sugar intake with children's weight status and showed that the main contributors to added sugar intake were sweets, including those made at home, processed cereals and SSBs. An association with overweight and obesity was found only among children and adolescents that exceeded the 10% upper recommended level of total calories intake from added sugars regardless of source (Magriplis et al. 2021). Carbohydrates and total fat intake were also associated with higher weight of Greek toddlers since a substantial percentage exceeded the adequate distribution range (Manios et al. 2008). Furthermore, recent evidence suggests that not all UPFs classified as NOVA 4 have the same effects on health, as shown by a recent systematic review and meta‐analysis in adults as well (Mendoza et al. 2024). It is also noteworthy that in another recent study, in a subgroup analysis, animal‐based products and artificially and SSBs were associated with an increased risk of multimorbidity of cancer and cardiometabolic diseases but on the other hand, associations were not observed with ultra‐processed bread and cereals or plant‐based alternatives (Cordova et al. 2023).

This study has several strengths. To our knowledge, this is the first study to evaluate dietary UPF proportion and their main food contributors among Greek children. As per the study results, this is an important area to examine since the effect of UPFs on bodyweight and potentially on health, possibly varies by type and amount of food group consumed. A crude NOVA 4 classification in one pool may lead to wrongful results, and based on data to date, this may be the reason why inconsistent findings on associations with weight are reported between studies. Most studies to date and to our knowledge have evaluated UPFs in total. The effects, however, on weight and health may vary by the nutritional profile of the food or by the amount consumed which may vary between countries. For example, the mean intake of added sugar in children from the United States was 12.4% of the total daily energy intake and SSBs (Ricciuto et al. 2022) were the main contributor whereas in Greece, the median was 9.4% and the main contributors were sweets and refined cereals (Magriplis et al. 2021). These were considered important variables to depict and were evaluated in this study, to provide more food‐specific population effects.

The results of our study should be interpreted with caution due to its retrospective nature. Inference on the time sequence of the association between UPF consumption and obesity cannot be made. The results of this study however, underline that even 10 years ago, UPF intake was relatively high among a large proportion of the population in Greece, which one can assume has further increased in the past years. The fact that an association was found between consumption of UPF higher than 62% as energy and obesity needs further investigation since only two children in our study had such a high UPF intake. Also, due to the small sample size, data were not stratified by age groups, with children and adolescents grouped in one category. Self‐reported data may also lead to errors, however information was validated with random measurements performed in a subsample of the population (*n* = 83 children). Overall, although there are discrepancies in the literature between the validity of self‐reported measures, a recent SR that evaluated the validity of measured versus self‐reported weight and height, found good agreement between measured and self‐reported weight and height based on intra‐class correlation coefficient (> 0.9) and Bland–Altman plots (Fayyaz et al. 2024). In the present study, measurements were taken by parents/guardians rather than the children themselves. To further decrease bias, missing data on weight or height were imputed as recommended following valid techniques (Little and Rubin 2020). It is also important to consider any potential NOVA categorisation misclassifications of food items, although this was highly minimised with the methodology followed.

## 5 Conclusion

This study found that the contribution of UPFs to total energy intake was relatively high for all children irrespective of weight status but there was no association between total UPF consumption and obesity. In addition to the total daily contribution of UPF, the type of UPFs in various populations should be determined and the specific effects of those highly consumed should be further evaluated. Public health awareness campaigns should focus on intakes of specific food groups and not only on total energy balance because, despite the lack of association with overweight and obesity seen in this study, the high consumption of UPFs and their low nutritional value, may lead to adverse public health consequences.

## Author Contributions

E.P. (Evgenia Petridi) contributed to study conception, design, data analysis and drafted the manuscript. E.M. is the supervisor of the data analysis of HNNHS and contributed to study conception and design, data analysis, supervised the work and provided critical input into the final draft of the manuscript. K.K., E.C. and E.P. (Elena Philippou) supervised the work and provided critical input into the final draft of the manuscript. A.Z. is the principal investigator of HNNHS, contributed to the concept and design of the work, and provided critical input into the final draft of the manuscript. All authors have read and agreed to the final version of the manuscript.

## Conflicts of Interest

The authors declare no conflicts of interest.

## Supporting information


Data S1.

## Data Availability

The data that support the findings of this study are available from the corresponding author upon reasonable request.
