# Supplementary material for: Ultra Processed Food Consumption in Children and Adolescents: Main Food Group Contributors and Associations With Weight Status
Source: Nutr Bull. 2025 Apr 1;50(2):278–89. doi: 10.1111/nbu.70001 (PMC12147056; doi:10.1111/nbu.70001)
Supplement: Supplementary file 1 — Data S1. [file NBU-50-278-s001.docx]

Supplementary methodology material

Missing data were imputed to reduce bias in the analysis performed that could result from simply excluding children with missing values. Missing at Random approach (MAR) was chosen since the missingness of data were related to other observed values (such as height and sex) but not to the missing values themselves. The process of the imputation using MAR included multiple steps. Firstly, indicator variables were created to identify the missing data and then two imputation models were created using simple regression models, one for weight and one for height, since these ae continuous variables. Age and sex were used as predictor variables in both models. Once imputation models were created, they were checked if the fit the data well. To do so, the model for weight, and then for height, were fitted to the observed data (age & sex), and the residuals were plotted against the fitted values, following logarithmic transformation on the response variable (model adjusted for age, sex and mean daily energy intake). These were cross- checked against the models without the imputations (Suppl Graph 1), after which final imputations were performed. A total of 5 imputations were created for each missing value, specifying rseed () option for reproducibility. Summaries of the observed, imputed and completed data were then derived and distributions graphs were constructed (Suppl Figure 2). Lastly Kolmogorov-Smirnov tests were used to test the hypothesis that the experimental/observed data are consistent with the theoretical (imputed) data. The combined K-S p value for weight was 0.750, in support of the null hypothesis. This was also tested for BMI, since it was the main explanatory variable addressed and requires both weight and height for its derivation. The combined K-S p-value for BMI was 0.218.

**Suppl Figure 1: Residuals of imputed weights and not imputed weights, versus fitted plot for age and sex**

**
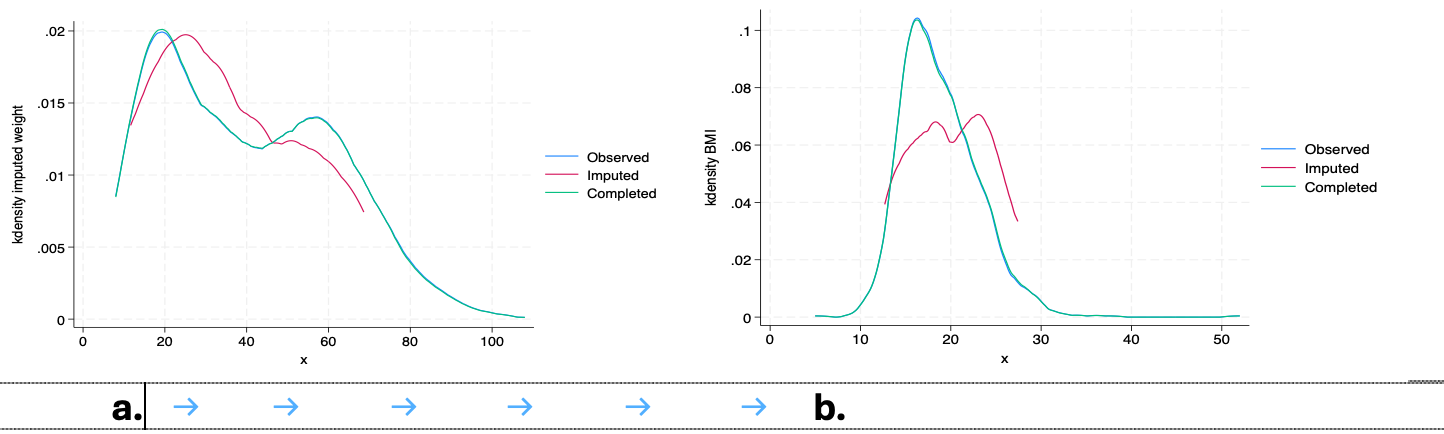
**

**Figure 2: Distributions of (a) weight and (b) BMI in the observed, imputed, and completed samples**
